# Functional Implications of Active N^6^-Methyladenosine in Plants

**DOI:** 10.3389/fcell.2020.00291

**Published:** 2020-04-29

**Authors:** Hongxiang Zheng, Simin Li, Xiansheng Zhang, Na Sui

**Affiliations:** ^1^Shandong Provincial Key Laboratory of Plant Stress, College of Life Sciences, Shandong Normal University, Jinan, China; ^2^State Key Laboratory of Crop Biology, College of Life Sciences, Shandong Agricultural University, Tai’an, China

**Keywords:** N^6^-methyladenosine, functional implications, plant, RNA function, stress response

## Abstract

N^6^-methyladenosine (m^6^A) is the most common type of eukaryotic mRNA modification and has been found in many organisms, including mammals, and plants. It has important regulatory effects on RNA splicing, export, stability, and translation. The abundance of m^6^A on RNA depends on the dynamic regulation between methyltransferase (“writer”) and demethylase (“eraser”), and m^6^A binding protein (“reader”) exerts more specific regulatory function by binding m^6^A modification sites on RNA. Progress in research has revealed important functions of m^6^A modification in plants. In this review, we systematically summarize the latest advances in research on the composition and mechanism of action of the m^6^A system in plants. We emphasize the function of m^6^A modification on RNA fate, plant development, and stress resistance. Finally, we discuss the outstanding questions and opportunities exist for future research on m^6^A modification in plant.

## Introduction

More than 150 RNA modifications have been identified as post-transcriptional regulatory markers in a variety of RNA species, including messenger RNA (mRNA), transfer RNA (tRNA), ribosomal RNA (rRNA), small non-coding RNA (snRNA), and long non-coding RNA (lncRNA), RNA methylation is one of the post-transcriptional modifications of RNA, and N^6^-methyladenosine (m^6^A) is the most common type of RNA methylation modification, accounting for more than 80% of RNA methylation modifications in organism. Current study suggests that the m^6^A modification plays an important role in RNA fate, such as RNA splicing (Liu et al., 2015, 2017; Haussmann et al., 2016; Lence et al., 2016; Xiao et al., 2016; Pendleton et al., 2017), RNA stability (Wang et al., 2014; Du et al., 2016; Mishima and Tomari, 2016; Huang et al., 2018), RNA export (Roundtree et al., 2017; Edens et al., 2019), 3′ untranslated region (UTR) processing (Ke et al., 2015; Bartosovic et al., 2017; Wei et al., 2018; Yue et al., 2018), translation (Zhou et al., 2015; Choi et al., 2016; Li et al., 2017; Shi et al., 2017), and miRNA processing (Alarcón et al., 2015a, b; Bhat et al., 2019). Although the presence of m^6^A was detected in mammals (Desrosiers et al., 1974; Wei et al., 1975; Schibler et al., 1977) and plants (Kennedy and Lane, 1979; Nichols, 1979) in the 1970s, it had not received much attention because it was considered to be “static” due to the method of detecting m^6^A sites. However, the discovery of the first m^6^A demethylase fat mass and obesity-associated protein (FTO) was an exciting development (Jia et al., 2011), as it demonstrated that the m^6^A modification process is dynamic and reversible in the cell. Subsequently, the methyl-RNA immunoprecipitation combined with RNA sequencing (MeRIP-Seq) method was established for identifying m^6^A modifications on mRNA in the transcriptome (Dominissini et al., 2012; Meyer et al., 2012). This method relies on the highly specific antibody of m^6^A to precipitate m^6^A and then involves high-throughput sequencing to reveal methylated transcripts (Dominissini et al., 2012; Meyer et al., 2012). This method revealed that the m^6^A site is not uniformly distributed over the mRNA: only some mRNAs have m^6^A sites, most of which are located near the stop codon and the 3′ UTR (Dominissini et al., 2012; Meyer et al., 2012). At the same time, m^6^A is highly dynamic, and the level of m^6^A varies greatly depending on the developmental stage (Dominissini et al., 2012; Meyer et al., 2012). These findings suggested that m^6^A modification may affect the fate and function of mRNA in cells. As more m^6^A-related enzymes are identified, the important biological functions played by m^6^A modification are being gradually unveiled. Although the study of m^6^A functions was mainly in animal systems, current studies shows that m^6^A modification also plays important role in regulating plant development (Zhong et al., 2008; Bodi et al., 2012; Shen et al., 2016; Hofmann, 2017; Růžička et al., 2017; Anderson et al., 2018; Arribas-Hernández et al., 2018; Chen et al., 2018; Scutenaire et al., 2018; Wei et al., 2018; Zhang et al., 2019; Zhou et al., 2019; Luo et al., 2020) and stress resistance (Martínez-Pérez et al., 2017; Anderson et al., 2018; Li et al., 2018; Miao et al., 2020).

Writers, erasers, readers are the core components of the m^6^A regulatory system. The writers and erasers are responsible for adding or removing m^6^A to the conserved sequence “RRACH” (where R = A/G, A is the modified m^6^A site, and H = A/C/U) (Dominissini et al., 2012; Schwartz et al., 2013; Li et al., 2014; Luo et al., 2014; Lence et al., 2016; Shen et al., 2016; Parker et al., 2020), respectively. The readers are responsible for binding m^6^A sites and play specific regulatory roles for modified-RNA. Writers, erasers, and readers form the basis of a complex regulatory network under the guidance of m^6^A modification. However, not all RNAs containing the “RRACH” sequence will have m^6^A added to them (Dominissini et al., 2012; Li et al., 2014). It is unclear how the writers and erasers selectively add or remove m^6^A on RNA sequences. Therefore, the discovery and functional studies of more m^6^A-related enzymes can help us to understand the mechanism of m^6^A regulation.

## The Main Components of the m^6^A System: Writers, Erasers, and Readers

Studies on m^6^A enzymes or novel functions have mainly focused on animal systems, while there have been few studies in plants, especially in crops. In mammals, m^6^A is produced by a methyltransferase complex consisting of MTase complex comprising methyltransferase-like 3 (METTL3) (Bokar et al., 1994), wilms’ tumor 1-associating protein (WTAP) (Agarwala et al., 2012), and methyltransferase-like 14 (METTL14) (Liu et al., 2014) and is removed by the action of the demethylases FTO (Jia et al., 2011) and α-ketoglutarate-dependent dioxygenase alkb homolog 5 (ALKBH5) (Zheng et al., 2013). This modification process is dynamic and reversible in the cell. The reader plays a specific regulatory role by recognizing the m^6^A modification site, which mainly includes the YTH (YT512-BHomology) domain-containing proteins YTHDC1/2 (DC1/2) (Bailey et al., 2017; Hsu et al., 2017; Roundtree et al., 2017; Zhang et al., 2010) and YTHDF1/2/3 (DF1/2/3) (Dominissini et al., 2012; Wang et al., 2014, 2015; Zhou et al., 2015; Shi et al., 2017), HNRNPA2B1 (Agarwala et al., 2012), and eukaryotic initiation factor 3 (eIF3) (Meyer et al., 2015). However, it should be emphasized that the core enzymes in the m^6^A system are highly conserved among different species, so studying the regulatory patterns of m^6^A in animals should also help us to explore its regulation in plants.

## Writers

**FIGURE 1:**
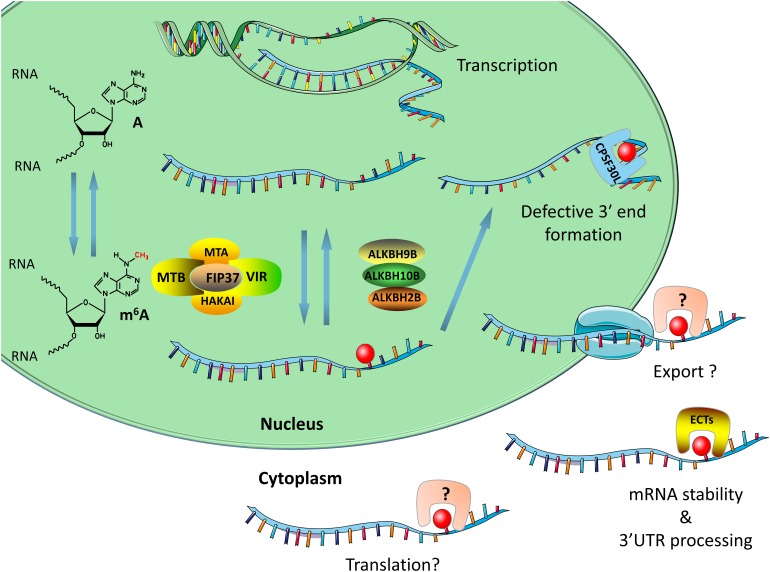
The main components of the m^6^A system in plants include writers, erasers, and readers. The writers consist of MTA, FIP37, MTB, HAKAI, and VIRILIZER. The demethylases are mainly ALKBH2, ALKBH9B, and ALKBH10B. The m^6^A binding proteins are mainly ECT family proteins and CPSF30, both of which contain a YTH domain. The writers and erasers are responsible for adding or removing m^6^A site on RNA. The readers interact with m^6^A-modified RNA and regulate RNA splicing, RNA stability, and 3′UTR processing. This figure was created using smart Servier Medical Art (https://smart.servier.com/).

In *Arabidopsis*, the METTL3 homolog MTA (At4g10760) is highly expressed in seeds, pollen microspores, and meristems. In loss-of-function mutants of T-DNA insertion, an embryonic lethal phenotype and m^6^A completion loss occur (Craigon et al., 2004). This is consistent with the phenomenon of METTL3 mutation in animals and yeast (Geula et al., 2015). Yeast two-hybrid assay and co-immunoprecipitation experiments showed that MTA protein interacts with the protein encoded by FIP37 (At3g54170) *in vitro* and *in vivo* (Zhong et al., 2008). FIP37 is a homolog of the selective cleavage protein WTAP in human and *Drosophila*. FIP37 expression patterns are similar to those of MTA. In addition, disruption of FIP37 by T-DNA insertion also results in an embryonic lethal phenotype with developmental arrest at the globular stage (Vespa et al., 2004; Růžička et al., 2017). MTB is a homolog of human METTL14, which has also been shown to be a part of the m^6^A methyltransferase complex (Liu et al., 2014). Experiments on RNA interference (RNAi) lines with inducible knockdown of MTB have shown that such knockdown leads to a nearly 50% reduction in m^6^A levels (Růžička et al., 2017). In addition, using the method of tandem affinity purification (TAP), VIRILIZER (KIAA1429 human homologous protein) (Schwartz et al., 2014) and E3 ubiquitin ligase HAKAI (HAKAI human homologous protein) were also found to be components of the *Arabidopsis* methyltransferase complex (Růžička et al., 2017). Inhibition of the expression of VIRILIZER and HAKAI resulted in a decrease in the level of m^6^A in *Arabidopsis* mRNA (Růžička et al., 2017). MTA, MTB, FIP37, VIRILIZER, and HAKAI are considered to be the main components of the m^6^A methyltransferase complexes in *Arabidopsis* system (Figure 1). In addition, the writers in the m^6^A system have also been reported in other plants. Knockout of OsFIP or OsMTA2 in rice significantly reduced the level of m^6^A, while no effect on total m^6^A levels was observed in the OsMTA1, OsMTA3, and OsMTA4 knockout lines (Zhang et al., 2019). This suggested that OsMTA2 and OsFIP are the main components of the m^6^A methyltransferase complex in rice (Zhang et al., 2019).

## Erasers

ALKBH9B (At2g17970) and ALKBH10B (At4g02940) have been shown to be active m^6^A demethylases concerning *Arabidopsis* system (Duan et al., 2017; Martínez-Pérez et al., 2017). ALKBH9B was the first m^6^A demethylase reported from *Arabidopsis*, which enables ssRNA to demethylate m^6^A *in vitro*. Moreover, ALKBH9B has a positive effect on viral abundance in plant cells. These findings indicate that methylation status plays an important role in regulating viral infection in *Arabidopsis* (Martínez-Pérez et al., 2017). Duan et al. (2017) also demonstrated that ALKBH10B-mediated demethylation of mRNA m^6^A affects the mRNA stability of key flowering time regulators, thereby affecting flower turnover. *In vitro* experiments and those involving transient transformation of tobacco showed that tomato SlALKBH2 can effectively remove m^6^A modification and reduce the m^6^A level *in vitro* and *in vivo* (Zhou et al., 2019). This indicates that tomato SlALKBH2 has m^6^A demethylation activity (Zhou et al., 2019).

## Readers

The member of the ECT family containing the YTH domain is the most important m^6^A binding protein in plants (Anderson et al., 2018; Arribas-Hernández et al., 2018; Scutenaire et al., 2018). Scutenaire showed that ECT2 binds to m^6^A via a tri-tryptophan pocket, and if these amino acids are mutated, ECT2 loses its m^6^A binding ability (Scutenaire et al., 2018). They also showed that *ect* mutants share phenotypes (defective trichomes) with *mta* mutants and FIP37-overexpressing transgenic lines, and the morphological changes in the *ect* mutant are the result of higher cell ploidy caused by intranuclear replication (Scutenaire et al., 2018), this result was consistent with the phenomenon observed by Arribas-Hernández et al. (2018). In addition, ECT2 improves the stability of m^6^A methylated RNAs transcribed from genes involved in trichome morphogenesis (Wei et al., 2018). This observation contrasts to the reported decrease in stability of RNAs caused by the binding of YTHDF proteins to this mark in animal systems (Du et al., 2016). However, a previous study by Shen in *Arabidopsis* found that m^6^A destabilizes a few transcripts in undifferentiated tissues (Shen et al., 2016). Thus, the mechanisms by which m^6^A regulates transcript stability have still not been completely clarified in any organism. In a study focused more on the morphological aspects of ECT proteins, including ECT2/3 and 4, it was shown that these proteins are intrinsically important for proper leaf morphogenesis, including trichome branching (Arribas-Hernández et al., 2018).

As described in a recent report, sequence analysis of m^6^A methyltransferase in 22 plants using *Arabidopsis* as a model plant revealed that, in higher plants, the number of m^6^A writers is greater than that in lower plants (Yue et al., 2019). This suggests that higher plants may require more precise mechanisms regulating m^6^A modification to cope with complex and variable environments (Yue et al., 2019).

Summarizing recent research, we can find that the key component genes of the m^6^A system are mainly concentrated in meristems and reproductive organs, and lower expression in tissues that stop differentiation and mature (Zhong et al., 2008; Hofmann, 2017; Růžička et al., 2017; Zhang et al., 2019; Zhou et al., 2019). This suggests that m^6^A modifications are more likely to occur on actively transcribed genes. Besides, m^6^A modifications are detected on mRNA, rRNA, tRNA, and sn(o) RNA in plant system (Li et al., 2014; Luo et al., 2014; Wan et al., 2015; Anderson et al., 2018; Parker et al., 2020).

## Effect of m^6^A Modification on RNA Function

The above main components of the m^6^A system above regulate the fate of RNA, by adding, removing, and binding m^6^A site on RNA. In mammals, m^6^A modification plays an important role in the regulation of RNA splicing (Liu et al., 2015, 2017; Haussmann et al., 2016; Lence et al., 2016; Xiao et al., 2016; Pendleton et al., 2017), RNA stability (Wang et al., 2014; Du et al., 2016; Mishima and Tomari, 2016; Huang et al., 2018), RNA export (Roundtree et al., 2017; Edens et al., 2019), 3′ UTR processing (Ke et al., 2015; Bartosovic et al., 2017; Wei et al., 2018; Yue et al., 2018), translation (Zhou et al., 2015; Choi et al., 2016; Li et al., 2017; Shi et al., 2017), and miRNA processing (Alarcón et al., 2015a, b; Bhat et al., 2019). On the contrary, much less is known about the function of m^6^A modification regulation of RNA on plant. Our understanding of how the m^6^A regulated RNA fate is limited to it’s an mRNA stabilizing (Shen et al., 2016; Hofmann, 2017; Wei et al., 2018) or 3′ UTR processing at specific genomic loci (Pontier et al., 2019) mark. The roles in regulating plant RNA export, RNA splicing, and translation remain unexplored. In addition, research on the effect of m^6^A modification on RNA has mainly focused on genetic interference, and there is no way to accurately predict the effect of m^6^A modification on RNA at the transcriptome-wide level. Only one or some of the effects of RNA due to changes in m^6^A modification can be identified.

## 3′ UTR Processing

In animal systems, m^6^A modification has been widely reported to regulate mRNA processing including RNA splicing (Liu et al., 2015, 2017; Haussmann et al., 2016; Lence et al., 2016; Xiao et al., 2016; Pendleton et al., 2017) and 3′ UTR processing (Ke et al., 2015; Bartosovic et al., 2017; Yue et al., 2018). For example, in *Drosophila*, m^6^A modification regulates the sex selection process by regulating alternative splicing of the sex determination factor Sex lethal (Sxl) pre-mRNA (Haussmann et al., 2016; Lence et al., 2016); In animal cells, METTL16 regulates the SAM synthetase gene *MAT2A* splicing process by regulating the m^6^A modification on *MAT2A* mRNA, thereby regulating regulate SAM homeostasis (Pendleton et al., 2017). YTH domain-containing protein YTHDC1 regulates the cleavage process by recognizing m^6^A on mRNA and recruiting the SR protein to its corresponding binding site (Xiao et al., 2016). Therefore, m^6^A is also considered to be a post-transcriptional regulator of mRNA splicing in animal systems.

In *Arabidopsis*, the methyltransferase VIRILIZER was found to be co-localized with the splicing factor SR34, but no abnormally spliced transcript was detected in the root of VIRILIZER mutant (Růžička et al., 2017). This suggests that m^6^A is not involved in large-scale splicing regulation of plant transcripts, which appears to contrast with the findings reported from animals (Xiao et al., 2016). Alternatively, variable splicing regulated by m^6^A occurs only on specific transcripts or specific tissues, but the level of this is below the limit of detection of the method used for analyzing it.

In mammals, m^6^A modification regulates alternative poly(A) sites (APA) during 3′ UTR processing (Ke et al., 2015; Bartosovic et al., 2017; Yue et al., 2018). Research by Ke et al. (2015) shows that higher m^6^A modification in the last exon may affect the usage of APA, while Bartosovic et al. (2017) further shows that m^6^A modification in the last exon regulates 3′ UTR length by regulating APA. A similar situation was found in plant systems. A recent study showed that the loss of methylation enzyme function of FIP37 resulted in a decrease in m^6^A modification (Shen et al., 2016) and the pair of spatially adjacent two genes (such as the pair AT4G30570/580 or AT1G71330/340) to form chimeric mRNA (Pontier et al., 2019). The m^6^A modification can assist in the polyadenylation of the first gene mRNA, thereby limiting mis-splicing to form chimeric mRNA (Pontier et al., 2019). However, this process requires the assistance of F30L, which is a protein comprising the typical m^6^A recognition protein domain YTH (Figure 1; Pontier et al., 2019). This suggested that the m-ASP (m^6^A-assisted polyadenylation) pathway ensures transcriptome integrity at rearranged genomic loci in plants (Pontier et al., 2019).

## mRNA Stability

How does m^6^A modification work in plant systems? The most recent report on this issue describes that m^6^A regulates plant growth and development by affecting mRNA stability. The lack of the *Arabidopsis* methyltransferase FIP37 results in reduced m^6^A modification on the mRNA encoded by SAM proliferation-related genes [WUSCHEL (WUS) and SHOOTMERISTEMLESS (STM)], and enhances its stability (Shen et al., 2016). Excessive accumulation of WUS and STM mRNA causes excessive proliferation of SAM (Shen et al., 2016). However, Duan et al. (2017) obtained results that differ from these findings. Specifically, in the functional deletion mutant of *Arabidopsis* demethylase ALKBH10B, m^6^A modification on the mRNA encoded by key genes regulating FT, SPL3, and SPL9 was increased, which reduced its stability, accelerated its degradation, and produced a delayed flowering phenotype (Hofmann, 2017). In addition, studies on the m^6^A reader ECT2 in plants have indicated that it plays an important role in regulating 3′ UTR processing in the nucleus and promoting mRNA stabilization in the cytoplasm (Figure 1; Wei et al., 2018). Loss of function of ECT2 accelerates the degradation of three ECT2-binding mRNAs involved in morphogenesis of the trichome, thereby affecting the branching of the trichome (Wei et al., 2018).

Although m^6^A modification may stabilize mRNA in plants, no consensus on this issue has yet been reached. In addition, after the modification of methylation of mRNA, m^6^A binding protein also plays an important role. Moreover, studies on the stability of mRNA by m^6^A modification have mostly focused on a single mRNA, and cannot explain the effect of m^6^A modification on mRNA stability across the transcriptome. In summary, m^6^A may have different effects on mRNA stability in different tissues or organs. It should be emphasized that m^6^A readers may play precise and complex regulatory roles by recognizing changes in m^6^A modification on mRNA.

## Plant Growth and Development

**FIGURE 2:**
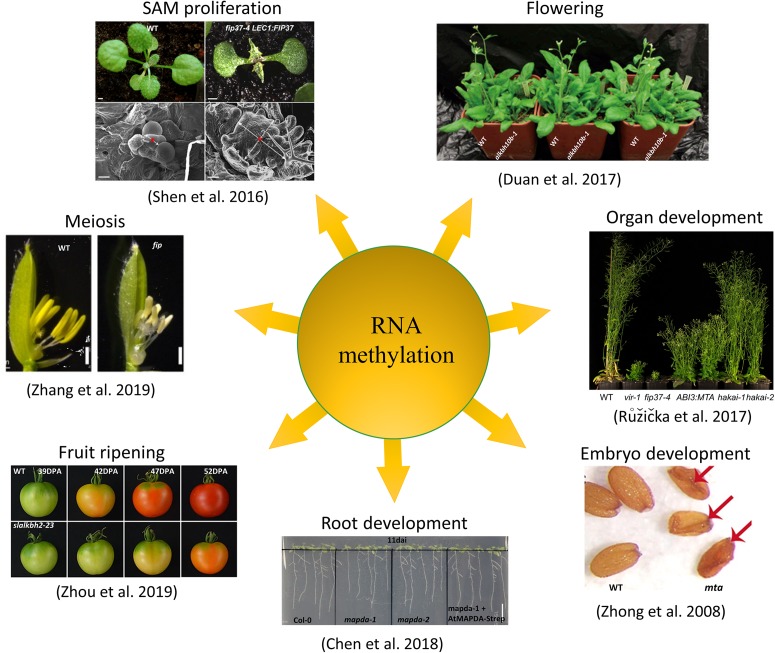
Functions of N^6^-methyladenosine in plants. In plant systems, m^6^A modification has been shown to be involved in regulating organ development, SAM proliferation, flowering, meiosis, embryo development, root development, and fruit ripening processes.

The mechanism of how m^6^A modification regulates the fate of plant RNA is still unclear. Previous studies have shown that the loss of function of any key component in the m^6^A system of writers, erasers, or readers can cause disorders in the m^6^A regulatory system, leading to abnormal growth and development (Figure 2). The lack or reduction of m^6^A writers, including MTA (Zhong et al., 2008; Anderson et al., 2018), MTB, FIP37 (Vespa et al., 2004), Virilizer (Růžička et al., 2017), and HAKAI (Růžička et al., 2017), results in a significant reduction in the overall level of m^6^A. This causes phenotypes including embryonic lethality, epidermal hair development abnormality, defective leaf sprouting, and excessive proliferation of vegetative shoot apical meristem. Moreover, loss of function of the eraser ALKBH10B results in leaf dysplasia and a delayed flowering phenotype in *Arabidopsis* (Hofmann, 2017). Several studies on m^6^A reader ECT family members have also comprehensively demonstrated the role of ECT protein in regulating *Arabidopsis* leaf and epidermal hair development (Arribas-Hernández et al., 2018; Scutenaire et al., 2018; Wei et al., 2018).

In addition, the role of m^6^A modification in regulating the growth and development of other plants has also begun to be discovered. In rice, the m^6^A writer OsFIP regulates the development of pollen microspores by directly mediating the addition of m^6^A to a group of threonine proteases and NTPase mRNA, and regulates its expression and splicing (Zhang et al., 2019). In addition, the complete loss of function of OsFIP leads to a decrease in the level of m^6^A modification and early degeneration of microspores at the vacuolated pollen stage (Zhang et al., 2019).

Summarizing current studies, we find that the core component of m^6^A in plant is mainly expressed in meristems, but at low levels in mature tissues and leaves. This suggests that the main regulatory mechanisms of m^6^A acting on plant growth and development are achieved by adding, removing, or recognizing m^6^A sites on transcripts that are particularly important for the growth and development of the above-mentioned organs and tissues. In addition, the use of genetic interference methods to study the function of m^6^A modification will lead to changes in the overall level of m^6^A modification, and produce unpredictable effects, we need a useful tool to exploring the functions of specific site m^6^A modifications on RNA.

## Function in Biotic Stress Adaptation

Plants have evolved a series of regulatory mechanisms in response to viral infections. These include sRNA (silencing based on small RNA) (Llave, 2010; Pumplin and Voinnet, 2013; Sharma et al., 2013), DNA methylation (Tirnaz and Batley, 2019), and RNA methylation (Martínez-Pérez et al., 2017). In animal systems, m^6^A modification has been reported to play an important role in regulating viral replication and the viral life cycle (Gokhale et al., 2016; Kennedy et al., 2016; Lichinchi et al., 2016a, b; Tirumuru et al., 2016). However, in plants, with the exception of the smaller group of DNA viruses, most viruses are RNA viruses. RNA viruses are hardly affected by DNA methylation because they do not have DNA during replication. As a widespread modification on RNA, m^6^A modification may have great potential in regulating plant anti-RNA virus infection.

In the *Arabidopsis* T-DNA insertion mutant of *alkbh9b*, the overall m^6^A level of viral RNA was found to be increased, and relative to the decrease in viral accumulation in the wild type, its resistance to alfalfa mosaic virus (AMW) was enhanced (Martínez-Pérez et al., 2017). It should be emphasized that ALKBH9B does not exhibit the ability to regulate cucumber mosaic virus (CMV) infection. This may be due to the fact that ALKBH9B can interact with the coat protein (CP) of AMV, but not with that of CMV (Martínez-Pérez et al., 2017). In addition, in tobacco, the level of m^6^A modification in tobacco is significantly reduced after infection with TMV (Li et al., 2018). This study suggests that m^6^A modification may represent a host regulatory mechanism for plants to respond to viral infections. Interestingly, in the genome of several single-stranded RNA plant viruses, ALKB containing a conserved domain has been identified (Bratlie and Drabløs, 2005; Van Den Born et al., 2008). This suggests that some plant viruses have evolved mechanisms to respond to host m^6^A system regulation.

## Abiotic Stress Process

In responding to environmental stress, m^6^A modification exhibits high sensitivity and complexity in the regulation of responses to heat stress, salt stress, and drought stress. Under salt stress, the m^6^A system enhances the stability of transcripts by adding m^6^A sites to salt-tolerant transcripts to regulate the salt tolerance process in *Arabidopsis* (Anderson et al., 2018). Under drought stress, the expression levels of the maize writer and reader members of the ALKBH10 family and ECT2 family were found to be increased, and the overall level of m^6^A modification in cells was decreased (Miao et al., 2020). In addition, in different genotypes of maize, m^6^A modifications were shown to be concentrated on different transcripts. This suggests that m^6^A modification is involved in the regulation of maize drought resistance and that there are different regulatory mechanisms in different genotypes of maize (Miao et al., 2020). Under heat stress conditions, the *Arabidopsis* reader ECT2 was found to respond to heat stress and relocate to stress granules (SGs) in the cell (Scutenaire et al., 2018; Wei et al., 2018). This process may result in the mRNA that binds to ECT2 relocalizing to stress particles under heat stress. Existing research suggests that the reader regulation of RNA is more direct and rapid than that by adding or erasing m^6^A sites on RNA, which relies on a writer and eraser. Regulation by a reader can be based on m^6^A modification on the original mRNA, and it can rapidly regulate the stress signal, especially in regulating short-term stress.

## Conclusion and Perspectives

At present, most m^6^A modification maps in plant systems was drawn by the m^6^A-seq method. However, there are some limitations to this approach, such as the need for a large number of samples, high requirements for antibody quality, and inability to accurately locate the position of m^6^A modifications on RNA. Although some improvements have been made to the resolution of m^6^A-seq, including m^6^A individual-nucleotide-resolution cross-linking and immunoprecipitation (miCLIP) (Linder et al., 2015), photo-crosslinking-assisted m^6^A-seq (PA-m^6^A-seq) (Chen et al., 2015), and m^6^A-cross-linking immunoprecipitation (m^6^A-CLIP) (Ke et al., 2015), but these improved methods still have not yet been tested in plants. In addition, m^6^A modifications are mainly concentrated in meristematic and reproductive organs, suggesting that m^6^A modifications are more likely to occur on actively transcribed genes. The sample size of these sites is often small, and the m^6^A-seq methods cannot accurately detect m^6^A modifications in tissues or cells and perform biological duplication. Therefore, for the development of new m^6^A detection methods, especially to reduce the sample size and improve detection accuracy, accurate identification of m^6^A modification at the cellular level is necessary.

Compared with detection methods based on NGS or PCR amplification, the technology of direct detection of m^6^A modification on RNA, including single-molecule real-time (SMRT) (Vilfan et al., 2013) and single-molecule nanoporous sequencing has great potential. Because PCR amplification is not required, direct detection-based methods do not produce base mismatches and PCR bias, and have the potential to detect multiple types of RNA modification at the same time. And only a lower sample starting amount is required. Ayub et al. have used α-hemolysin (αHL) nanopore sequencing to distinguish between modified and unmodified bases in RNA, including m^6^A and 5-methylcytosine (m^5^C) (Ayub and Bayley, 2012). Especially in recent years, nanopore sequencing technology has developed rapidly. Garalde et al. have developed a method for highly parallel direct RNA sequencing on Highly parallel direct RNA sequencing on an array of nanopores (Garalde et al., 2018). Parker et al. used nanopore sequencing technology to map the m^6^A modification in *Arabidopsis thaliana*, and revealed the complexity of m^6^A dynamic modification during mRNA processing (Parker et al., 2020). Therefore, we believe that nanopore sequencing is very suitable for studying small molecule samples and has the potential to accelerate the study of biological functions of modifications on RNA.

The m^6^A enzyme plays a fundamental role in the m^6^A regulatory system. However, the number of m^6^A enzymes found to date in plants is small relative to the number in animals, and no homolog of the major demethylase FTO in animals has been found. Only one demethylase of the ALKBH family was discovered (Hofmann, 2017; Martínez-Pérez et al., 2017; Zhou et al., 2019), and it is unclear whether ALKBH family protein can complete the removal of the m^6^A site on the mRNA. Therefore, it is also very important to find more key components of the m^6^A system in plants. In addition, it is not clear how writers and erasers selectively add or remove m^6^A on RNA, which may be related to the special secondary structure of RNA. Cryo-electron microscopy and molecular imaging may help to explore the process of m^6^A selective modification.

The main way to explore the function of m^6^A modification is still through genetic interference. However, the impact of adding or removing any key component of the m^6^A system on plants may be far more than we are concerned about. Therefore, the development of RNA methylation without changing the nucleotide sequence and the overall m^6^A modification level may be a major development regarding m^6^A for exploring the m^6^A function in the future. The CRISPR–Cas9 technology is rapidly evolving and has enabled accurate genome editing, including targeted DNA cleavage, repair, direct base editing, and site-specific epigenome editing. Recently, researchers have used a similar method to fuse m^6^A writers or erasers with Cas protein, and under the guidance of sgRNA and PAMer, edit the m^6^A modification on specific mRNA in the cell (Wei and He, 2019). This method of editing m^6^A did not change the nucleotide sequence and the overall m^6^A modification level (Wei and He, 2019). This method provides a new tool for studying the biological function of m^6^A modification and makes it possible to edit m^6^A at a specific site to improve crop quality.

## Author Contributions

HZ and SL prepared the manuscript. NS and XZ conceptualized the idea and revised the manuscript. All authors read and approved the final manuscript.

## Conflict of Interest

The authors declare that the research was conducted in the absence of any commercial or financial relationships that could be construed as a potential conflict of interest.
